# Identification of urban particulate matter-induced disruption of human respiratory mucosa integrity using whole transcriptome analysis and organ-on-a chip

**DOI:** 10.1186/s13036-019-0219-7

**Published:** 2019-11-15

**Authors:** Junhyoung Byun, Boa Song, Kyungwoo Lee, Byoungjae Kim, Hae Won Hwang, Myung-Ryul Ok, Hojeong Jeon, Kijeong Lee, Seung-Kuk Baek, Sang-Heon Kim, Seung Ja Oh, Tae Hoon Kim

**Affiliations:** 1 Department of Otorhinolaryngology-Head & Neck Surgery, College of Medicine, Korea University, 73, Inchon-ro, Seongbuk-gu, Seoul, 02841 Korea; 2 Center for Biomaterials, Korea Institute of Science and Technology, 5, Hwarang-ro 14-gil, Seongbuk-gu, Seoul, 02792 Republic of Korea; 3 Neuroscience Research institute, Korea University, College of Medicine, Seoul, Republic of Korea; 4 Department of Biomedical Engineering, University of Science and Technology, Daejon, Republic of Korea

**Keywords:** Cell integrity, Urban particulate matter, Organ-on-a-chip, Human nasal mucosa

## Abstract

**Background:**

Exposure to air particulate matter (PM) is associated with various diseases in the human respiratory system. To date, most in vitro studies showing cellular responses to PM have been performed in cell culture using a single cell type. There are few studies considering how multicellular networks communicate in a tissue microenvironment when responding to the presence of PM. Here, an in vitro three-dimensional (3D) respiratory mucosa-on-a-chip, composed of human nasal epithelial cells, fibroblasts, and endothelial cells, is used to recapitulate and better understand the effects of urban particulate matter (UPM) on human respiratory mucosa.

**Results:**

We hypothesized that the first cells to contact with UPM, the nasal epithelial cells, would respond similar to the tissue microenvironment, and the 3D respiratory mucosa model would be a suitable platform to capture these events. First, whole transcriptome analysis revealed that UPM induced gene expression alterations in inflammatory and adhesion-related genes in human nasal epithelial cells. Next, we developed an in vitro 3D respiratory mucosa model composed of human nasal epithelial cells, fibroblasts, and endothelial cells and demonstrated that the model is structurally and functionally compatible with the respiratory mucosa. Finally, we used our model to expose human nasal epithelial cells to UPM, which led to a disruption in the integrity of the respiratory mucosa by decreasing the expression of zonula occludens-1 in both the epithelium and endothelium, while also reducing vascular endothelial cadherin expression in the endothelium.

**Conclusions:**

We demonstrate the potential of the 3D respiratory mucosa model as a valuable tool for the simultaneous evaluation of multicellular responses caused by external stimuli in the human respiratory mucosa. We believe that the evaluation strategy proposed in the study will move us toward a better understanding of the detailed molecular mechanisms associated with pathological changes in the human respiratory system.

## Background

Recently, air pollution caused by sand, dust, smog, and urban particulate matter (UPM) has become a significant concern as it leads to a variety of health problems [1, 2]. UPM is a complex mixture that contains carcinogenic chemicals, including inorganic ions, metals, and polycyclic aromatic hydrocarbons (PAHs) [3]. Inhalation of UPM induces cardiovascular diseases, neoplastic disorders, and various respiratory diseases such as lung cancer, chronic obstructive pulmonary disease (COPD), asthma, and rhinitis [4–7]. These diseases develop when UPM penetrates the human respiratory mucosa and accumulates in target tissues [8]. However, the mechanism by which UPM infiltrates into the respiratory mucosa and induces genetic alteration is still unknown.

Human respiratory mucosa is the first point of contact for hazardous materials such as inhaled bacteria, viruses, pollen, and air pollutants, and thus, acts as a physical and immune barrier to harmful environmental factors [9]. It consists of the superficial epithelium, submucosal glands, subepithelial stromal cells, and endothelial cells. The cells adjacent to the mucosa maintain their structure and function [9]. The superficial epithelium is composed of a surface gel layer, sol layer, and apical junctional complexes (AJCs) that bind the adjacent cells [10]. AJCs regulate the passage of ions, water, and other molecules through the paracellular pathway, maintaining cell integrity. AJCs consist of junctional proteins and adherens proteins, including occludins, claudins, and zonula occludens-1 (ZO-1) [11, 12]. Changes in the protein levels within AJCs by allergens, toxins, or microorganisms disrupt cell integrity, increase intracellular permeability, and promote pathogen penetration, thereby contributing to the development of various mucosal diseases such as Crohn’s disease, allergic conjunctivitis, allergic rhinitis, and asthma [12–15]. In the subepithelial mucosa, loss of endothelial integrity may cause an increase in vascular permeability, which leads to a potential increase in extracellular edema [16]. This phenomenon occurs following the release of various stromal cytokines from the adjacent cell network [17].

Three-dimensional (3D) tissue culture platforms, such as cancer-on-a-chip and immune organs-on-a-chip, have been developed to mimic the in vivo cell culture conditions for better prediction of cellular behaviors [18]. As organ-on-a-chip technology has gained immense interest, there have been vast technological advancements in this field [19]. Although there have been various studies using organ-on-a-chip, the adverse effects of human respiratory mucosa exposure to air pollutants in vitro, by utilizing the organ-on-a-chip, have not been reported [20]. While most of the previous studies have focused on single-cell components during the development of the 3D platform, for a more complete understanding of human physiology, it is necessary to capture the final readouts derived from multicellular communications occurring in the tissue [20, 21]. Here, we propose an evaluation strategy for screening the effects of air pollutants on the human respiratory mucosa using the organ-on-a-chip. First, we developed an in vitro respiratory mucosa-on-a-chip consisting of three tissue layers mimicking the epithelium, extracellular matrix, and endothelium. Next, we used the in vitro respiratory mucosa-on-a-chip to investigate the effects of air pollutant exposure on the human tissue. It was observed that UPM disrupts both epithelial and endothelial tissue integrity, and the disruptions resulted from multicellular communications, as evaluated using our model system. Moreover, UPM-mediated disruption of epithelial tissue integrity was associated with alterations in the expression of genes related to cell adhesion and inflammation, which were observed using whole transcriptome analysis. Therefore, our evaluation strategy using the 3D respiratory mucosa platform with the multicellular components is also suitable for analyzing the complicated responses to external pathogens.

## Results

### Evaluation strategy for screening the effects of air pollutants on the human respiratory mucosa by using organ-on-a-chip

**Fig. 1:**
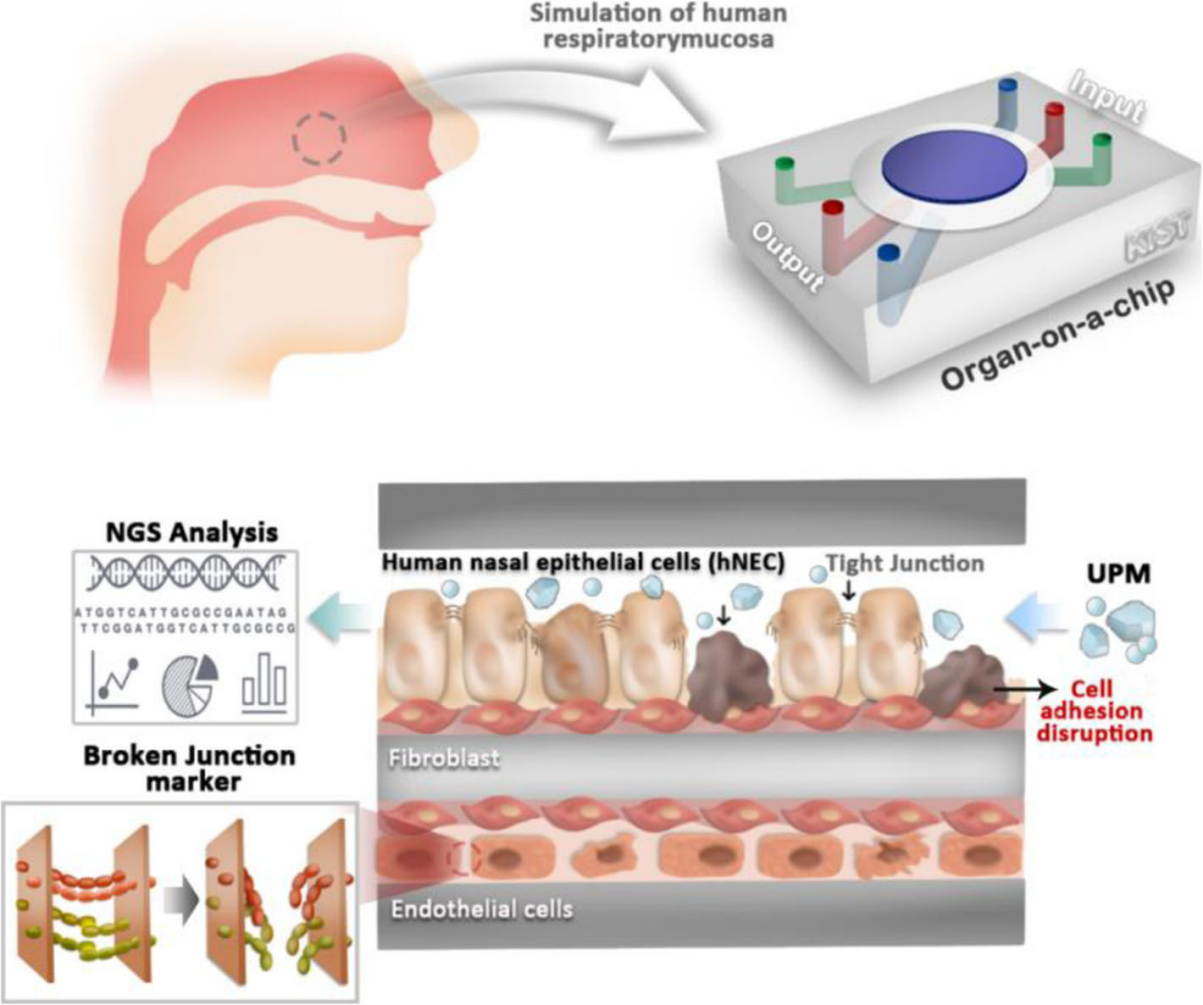
Evaluating the effects of air pollutants on human respiratory mucosa using organ-on-a-chip. The proposed pathway for screening for the effects of air pollutants on the human respiratory mucosa in shown. The respiratory mucosa-on-a-chip consists of three component layers: human nasal epithelial cells (hNEC), fibroblasts, and endothelial cells. Once the hNEC were exposed to the UPM, cell junctions were disrupted because of alterations in gene expression. Subsequently, it induced endothelial cell pro-inflammatory responses, thereby disrupting endothelial junction proteins

Figure 1 shows the proposed pathway for screening the effects of air pollutants on human respiratory mucosa. The respiratory mucosa-on-a-chip consisted of three components: an epithelial cell layer, a fibroblast layer, and an endothelial cell layer. Once the epithelial cell was exposed to the UPM, epithelial integrity was disrupted because of alterations in the expression of genes related to cell adhesion and inflammation. Subsequently, it induced pro-inflammatory responses in the endothelial cells, thereby disrupting endothelial integrity.

### UPM affects the three cell types in the human respiratory mucosa

**Fig. 2:**
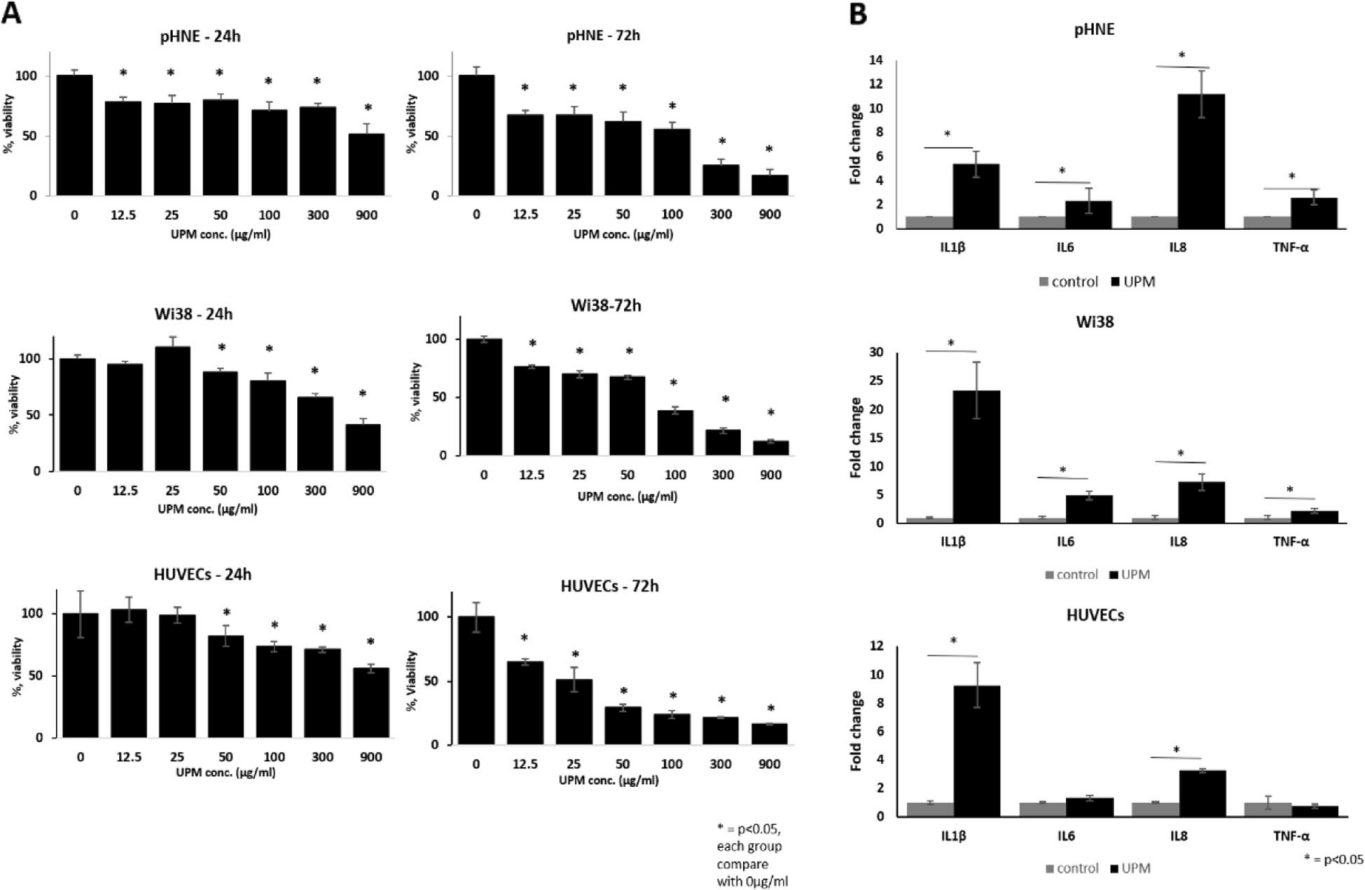
Effects of UPM on the three different cell types in human respiratory mucosa. **a** Cytotoxic analysis of human nasal epithelial cells (pHNE), fibroblast cells (Wi38), and human umbilical vein endothelial cells (HUVEC) were treated with the indicated concentrations of UPM for 24 and 72 h. **b** Transcriptional analysis of pro-inflammatory cytokines, such as IL-1β, IL-6, IL-8, and TNF-α in human respiratory mucosa cells were treated with 100 μg/mL UPM. The results presented in the graph were from three independent experiments. The error bars indicate mean ± SEM. *p < 0.05

UPM induces various inhalation-related diseases. However, there is no systemic analysis for understanding how these multicellular components communicate in vitro. To evaluate the effects of UPM on human respiratory mucosa, a viability assay was conducted using the human cellular system components separately including the primary human nasal epithelial cells (pHNE), human lung fibroblasts (WI-38), and human umbilical vein endothelial cells (HUVEC). Cells were incubated with UPM for 24 h or 72 h, followed by a cell viability assay. As shown in Fig. 2a, the viability of pHNE, WI-38, and HUVEC significantly decreased after 24 h or 72 h of treatment with UPM. Interestingly, pHNE were more sensitive to UPM than were the other cells under the same conditions. At a lower concentration, i.e., below 50 μg/mL, other cell lines showed less than 5% cell death, while the pHNE showed approximately 20% cell death. All three cell types showed approximately 50% viability at the highest concentration of UPM, i.e., 900 μg/mL. The pHNE and other cells showed a relatively similar viability trend after 72 h. These results indicate that UPM has a cytotoxic effect on cell viability with different sensitivity among the cell types. Then, we investigated the expression of pro-inflammatory cytokines, such as IL-1β, IL-6, IL-8, and TNF-α, by PCR. Each cell type was cultured in 6-well plates, treated with UPM for 24 h, RNA was isolated, and reverse-transcription of the RNA was performed to obtain cDNA. As shown in Fig. 2b, pro-inflammatory cytokine mRNA expression of respiratory mucosal cells significantly increased after 24 h of treatment with UPM (100 μg/mL). Specifically, IL-6 levels were 11 times higher for UPM-treated pHNE than for untreated controls. Wi38 and HUVEC had dramatically higher levels of IL-1β following UPM treatment. In HUVEC, TNF-α and IL-6 levels were unchanged. Thus, the various respiratory mucosa cellular components showed distinctive responses toward UPM with different durations of exposure.

### Whole transcriptome analysis of primary human nasal epithelial cells exposed to UPM

**Fig. 3:**
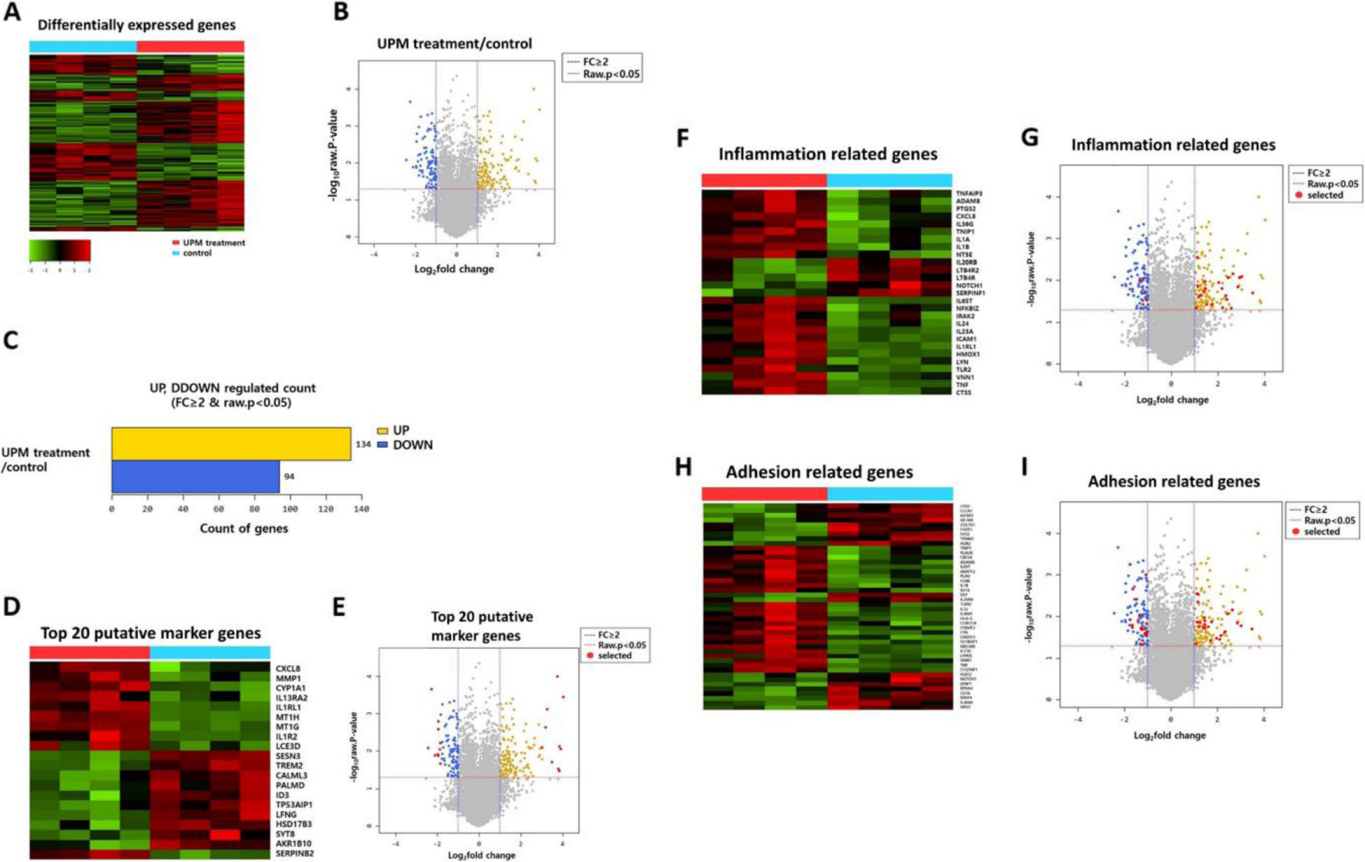
Whole transcriptome analysis of primary human nasal epithelial cells exposed to UPM. **a** Heat map and **b** volcano plot of all genes are shown. Those with a *p*-value < 0.05 and a fold-change (FC) of 2 or more are highlighted. **c** The number of genes with a *p*-value < 0.05 and a FC of 2 or more is shown in the bar graph. The top 20 putative marker genes were analyzed in **d** a heatmap and **e** volcano plot. The red dots on the volcano plot are selected genes by DEG analysis. The heatmap and volcano plots of the genes related to **f** and **g** inflammation and **h** and **i** adhesion; *n* = 4

To further investigate the mechanisms underlying the reduced viability following UPM treatment, the whole gene expression profile of pHNE in response to UPM treatment was investigated. pHNE from four individuals were exposed to 100 μg/mL UPM for 24 h, and responses were analyzed using RNA-Seq. Trimmed data were created by using the Trimmomatic program to remove adapter sequences and bases that did not exceed the baseline levels in the transcriptome raw data. The trimmed data were then mapped to the human genomic DNA reference, the UCSC hg19 assembly, by using HISAT2 software. A heatmap display showing gene expression of the control and UPM-treated primary human nasal epithelial cell groups is shown in Fig. 3a. Analysis of the differentially expressed genes (DEGs) was performed using the fragments per kilobase of exon per million mapped reads (FPKM) method. FPKM, which is a standardized value, considers the number of reads, transcript length, and depth of the application. DEGs were considered significant when the *p*-value was ≤0.05 and a fold change of > 2 was obtained. The expression level of all genes is shown as a volcano plot (Fig. 3b). As shown in Fig. 3c, 134 genes were upregulated, and 94 genes were downregulated in the UPM-exposed group relative to the control group. The top 20 DEGs are shown in Fig. 3d and e as a heatmap and volcano plot. Table 1 shows the top 10 upregulated genes and the top 10 down-regulated genes relative to the control.

**Table 1:** The top 10 differentially expressed genes when comparing urban particulate matter treatment and the untreated control group

Gene symbol	Description	RNA fold-change	*P*-value
Upregulated genes			
*MT1H*	metallothionein 1H	16.13	0.0004
*IL13RA2*	interleukin 13 receptor subunit alpha 2	14.92	0.009
*MMP1*	matrix metallopeptidase 1	14.31	0.008
*LCE3D*	late cornified envelope 3D	14.17	0.034
*IL1R2*	interleukin 1 receptor type 2	13.79	0.03
*MT1G*	metallothionein 1G	13.39	0.0001
*CXCL8*	C-X-C motif chemokine ligand 8	11.20	0.020
*SERPINB2*	serpin family B member 2	9.44	0.0008
*CYP1A1*	cytochrome P450 family 1 subfamily A member 1	8.97	0.002
*IL1RL1*	Interleukin 1 receptor-like 1	8.00	0.008
Downregulated genes			
*CALML3*	calmodulin like 3	−5.46	0.008
*SESN3*	sestrin 3	−4.82	0.0002
*ID3*	inhibitor of DNA binding 3, HLH protein	−4.31	0.013
*PALMD*	palmdelphin	−3.94	0.012
*AKR1B10*	aldo-keto reductase family 1 member B10	− 3.86	0.013
*LFNG*	LFNG O-fucosylpeptide 3-beta-N-acetylglucosaminyltransferase	−3.86	0.003
*TREM2*	triggering receptor expressed on myeloid cells 2	−3.83	0.002
*HSD17B3*	hydroxysteroid 17-beta dehydrogenase 3	−3.70	0.009
*TP53AIP1*	tumor protein p53 regulated apoptosis inducing protein 1	−3.64	0.006
*SYT8*	synaptotagmin 8	−3.59	0.022

Further, the functions of the identified DEGs were analyzed. Genes related to inflammation and adhesion were found to be significantly affected by UPM. A total of 27 genes in the inflammation category were altered when compared with the controls and are described by a heatmap and a volcano plot (Fig. 3f and g). The top five genes in the inflammation category were *CXCL8, IL1RL1, PTGS2, ICAM1,* and *ADAM8*. A heatmap and volcano plot were used to display adhesion-related genes, and the expression of 44 genes was found to be altered relative to the controls (Fig. 3h and i). The top five genes in the adhesion category were *CXCL8, AKAP12, ICAM1, ADAM8,* and *IL1B*. The detailed subcategories are described in Table 2. These results indicate that UPM can affect the nasal epithelium by altering the expression of genes related to inflammation and adhesion, and this may alter the tissue microenvironment.

**Table 2:** Gene ontology classification for selected categories

Category	Gene symbol
Inflammation	*CXCL8, IL1RL1, PTGS2, ICAM1, ADAM8, IL36G, IL1B, HMOX1, IL23A, TNFAIP3, IL24, IL1A, IRAK2, TLR2, IL6ST, TNIP1, TNF, CTSS, NFKBIZ, NT5E, LYN, VNN1, LTB4R, SERPINF1, IL20RB, NOTCH1, LTB4R2*
Adhesion	*CXCL8, AKAP12, ICAM1, ADAM8, IL1B, IL23A, HLA-G, LYPD5, IL32, TGM2, NRCAM, PLAUR, CORO1A, C1QTNF1, IL6ST, PLAU, TNIP1, TNF, SH3KBP1, FERMT2, FLNB, CARD11, NT5E, LYN, VNN1, MCAM, ICAM4, FLRT2, SFRP1, CD36, COL7A1, IL20RB, NOTCH1, FGFR3, EPHA4, TENM2, IGFBP2, AGR2, DST, WNT4, CLCA2, LY6D, FAT2, VAV3*

### Generation and reconstitution of 3D human respiratory mucosa-on-a-chip

**Fig. 4:**
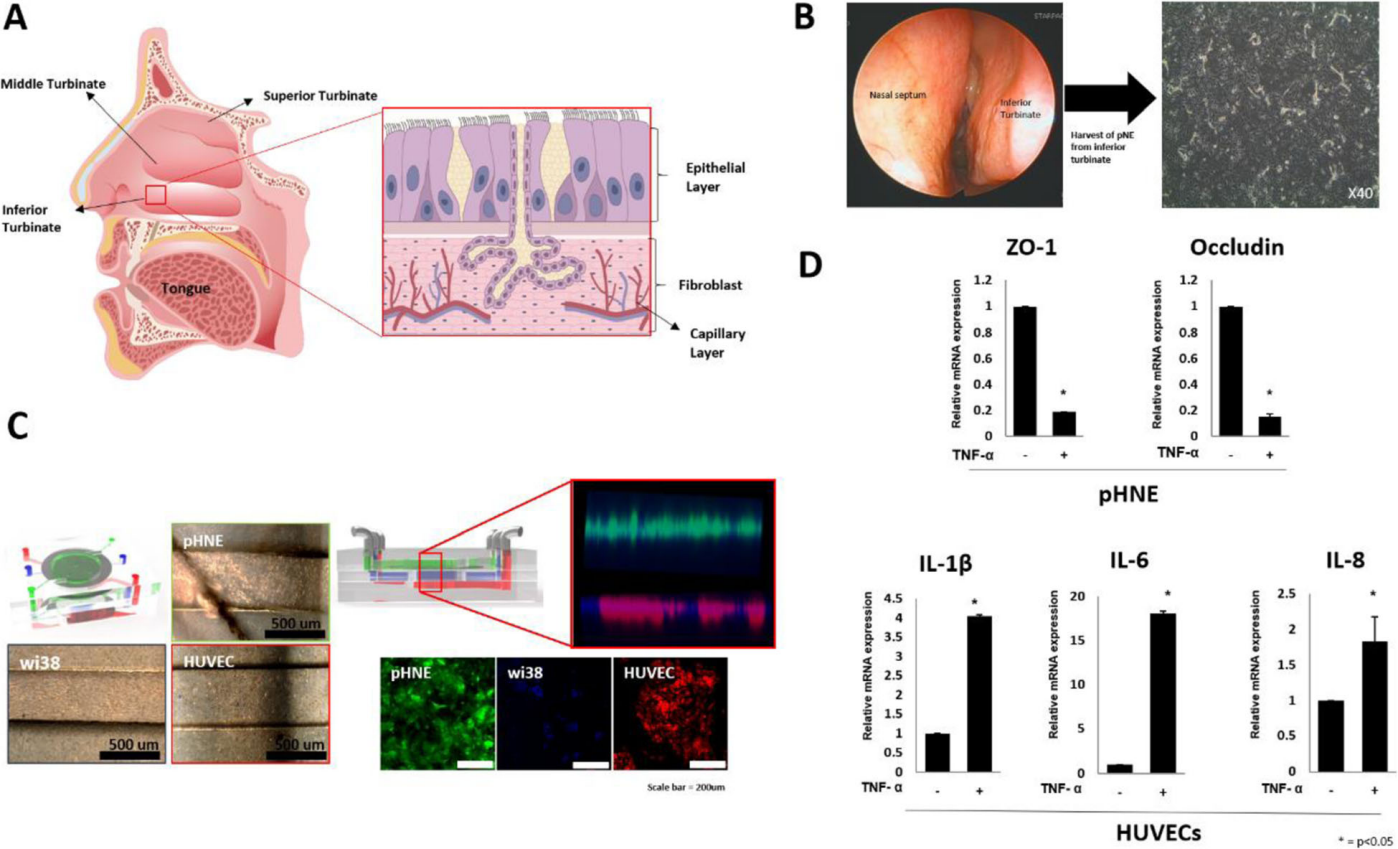
Generation and reconstitution of human respiratory mucosa-on-a-chip. **a** The schematic diagram of the complexity of the human nasal mucosa. **b** Primary nasal epithelial cells from the inferior turbinate were harvested and cultured. **c** Isolated pHNE, WI38, and HUVEC were seeded in the epithelial layer, fibroblast layer, and endothelial layer of the chip, respectively. **d** Transcriptional expression of epithelial junction proteins (ZO-1 and occludins) in epithelial cells and inflammatory cytokines (IL-1β, IL-6, and IL-8) in endothelial cells was examined at 24 h after TNF-α treatment. The results presented in the graph were from three independent experiments. The error bars indicate mean ± SEM. *p < 0.05

**Fig. 5:**
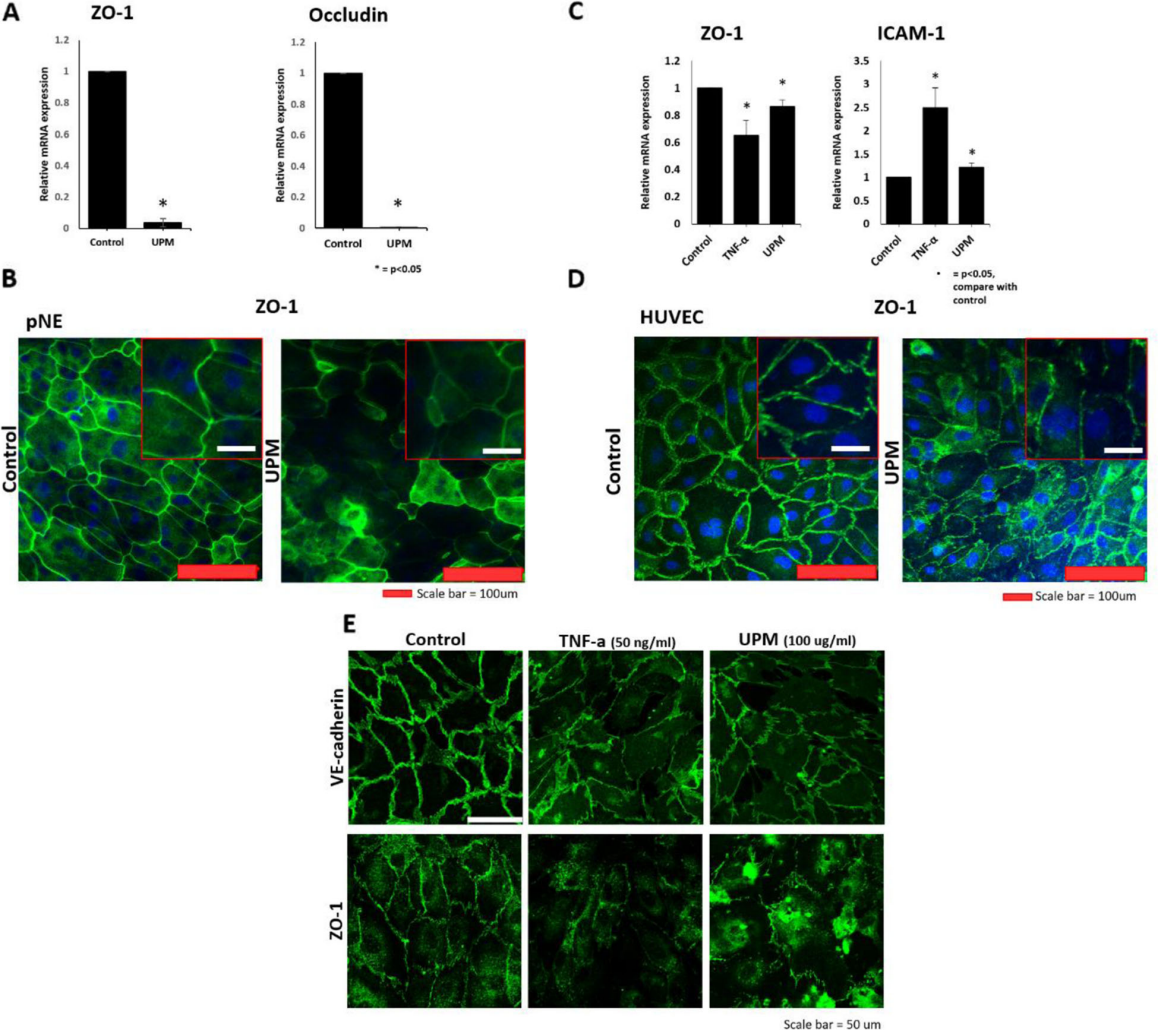
Air pollutant effects on human respiratory mucosa using in vitro respiratory mucosa-on-a-chip When UPM (100 μg/ml) was treated to the epithelial layer, the expression of epithelial junction proteins was examined **a** in epithelial layer by real-time PCR; ZO-1, occludin and **b** in epithelial cells by immunofluorescence; ZO-1. **c** The mRNA expression of ZO-1, ICAM-1 treated with UPM or TNF-α in HUVEC was compared using human respiratory mucosa-on-a-chip. **d** ZO-1 was detected in HUVEC by immunofluorescence. **e** HUVEC was treated with TNF-a or UPM, and ZO-1 and VE-cadherin were compared by immunofluorescence. The results presented in the graph were from three independent experiments; the error bars indicate mean ± SED. *P < 0.05

As UPM alters the expression of adhesion and inflammation-related genes in human nasal epithelial cells, we hypothesized that this could trigger changes in the tissue microenvironment. Thus, further studies were conducted to understand the alterations in the remaining cellular components, including endothelial cells in nasal tissue, which has a highly complex structure (Fig. 4a). Therefore, a 3D tissue platform containing the multicellular components was developed in this study. To mimic the structure of the human nasal mucosa, the 3D platform was designed with three separate layers of tissue, the epithelial, fibroblast, and endothelial layers. The human nasal inferior turbinate mucosa was harvested from suitable patients, and pHNE were isolated (Fig. 4b). As shown in Fig. 4c, the nasal tissue structure was assembled by seeding pHNE (1 × 10^6^ cells/ml), WI-38 (2 × 10^6^ cells/ml), and HUVEC (2 × 10^6^ cells/ml) on the top, middle, and bottom membranes, respectively. We confirmed cell attachment on the membrane layer by capturing images of each channel. The cell attachment was also traced by a fluorescent tagging system. Cells were seeded on the membrane of each layer. Utilizing the 3D platform, we tested the responses of human endothelial cells to TNF-α treatment (Fig. 5c). As expected, we found an increase in the mRNA expression of the pro-inflammatory mediators, IL-1β, IL-6, and IL-8, in the HUVEC. Further, we observed changes in the human nasal epithelium after treatment with TNF-α. TNF-α has been shown to regulate junction proteins. Therefore, we directly checked the expression levels of the tight junction proteins, including ZO-1 and occludins, and found decreased mRNA levels of both. Thus, the human respiratory mucosa-on-a-chip, mimicking the architecture and function of the human tissue, was successfully constructed. We also concluded that TNF-α induces changes in the multicellular components of the nasal tissue microenvironment, and we could simultaneously capture these events by utilizing the respiratory mucosa-on-a-chip. Thus, the 3D human respiratory mucosa-on-chip is suitable for studying the effects of air pollutants on respiratory mucosa.

### Air pollutant effects on human respiratory mucosa using in vitro respiratory mucosa-on-a-chip

The 3D respiratory mucosa-on-a-chip was used to test the hypothesis that UPM affects the epithelium, ultimately triggering a change in the tissue microenvironment. First, the UPM (100 μg/mL) was applied to the epithelial layer in the respiratory mucosa-on-a-chip. Then, epithelial and endothelial changes were analyzed. As shown in Fig. 3h and i, UPM induces alterations in the expression of genes related to cell adhesion and tight junctions. Thus, the junction markers from the epithelial layer were directly tested by RT-PCR. ZO-1 and occludins are junction proteins expressed in epithelial cells. The tissue integrity mediated by these junctional proteins is critical in normal physiology, and loss of function has been shown in various diseases. As shown in Fig. 5a, the expression of ZO-1 and occludins significantly decreased with the treatment of UPM. This was also confirmed by observing the tissue integrity mediated by ZO-1. Together, the expression of ZO-1 was decreased and tissue integrity was disrupted by UPM (Fig. 5b).

Further, the endothelial layer was analyzed to observe any changes triggered by the alterations of the epithelium. Intracellular adhesion molecule 1 (ICAM-1) is known to be expressed in endothelial cells and plays a pivotal role in inflammatory responses. Endothelial cells overexpress ICAM-1 during inflammation to capture immune cells, such as neutrophils. Since we hypothesized that UPM facilitates changes in the tissue microenvironment, which include epithelial cell changes by inducing inflammatory responses, we verified whether these changes affect the expression of ICAM-1 in the endothelium. As shown in Fig. 5c, the mRNA expression of ICAM-1 increased in endothelial cells upon exposure of the chip epithelial cells to UPM. Moreover, ZO-1 expression in endothelial cells decreased. Next, we investigated the cell junctions to look for disruptions in the HUVEC (Fig. 5d). Since vascular endothelial cadherin (VE-cadherin) is important for vascular permeability, we hypothesized that the UPM-induced changes in the tissue microenvironment may also affect vessel integrity. As shown in Fig. 5e, UPM treatment directly or indirectly disrupted VE-cadherin-mediated junctions, and this morphological change was similar to the phenotype induced by TNF-α. Thus, the disrupted integrity of the epithelium, with altered gene expression induced by UPM treatment, promotes immune responses and disrupted vessel integrity in the endothelial layer on the chip.

## Discussion

Air pollution has been shown to cause severe respiratory diseases, including cancers [22, 23]. However, the underlying detailed molecular mechanism associated with intercellular communication is still unclear. Once the human body is exposed to a pollutant, it triggers signaling cascades involving various cell types [22–24]. Therefore, it is important to study communication among different cell types to understand the underlying mechanism causing disease. Most of the previous in vitro studies that examined the effects of air pollution primarily focused on analyzing the responses of a single cell type [2, 4, 23, 25]. Therefore, it is important to understand the various signaling cascades within the tissue microenvironment.

In this study, we examined a strategy for evaluating air pollution-triggered multicellular responses by utilizing respiratory mucosa-on-a-chip. First, we generated an in vitro respiratory mucosa-on-a-chip by assembling human nasal epithelial cells, fibroblasts, and endothelial cells on three distinct membrane layers to mimic the human nasal architecture. Then, we confirmed that UPM disrupts the integrity of the human nasal epithelium, which subsequently showed reduced expression of the junctional protein, ZO-1. We also showed that UPM applied to the human nasal epithelium induces pro-inflammatory responses in endothelial cells in which an adhesion molecule, ICAM-1, is upregulated. Finally, we demonstrated that endothelial integrity is also affected by the treatment of epithelial cells with UPM. We exposed epithelial cells on-a-chip to UPM, which could either directly affect endothelial cells by going through disrupted epithelial junctions or it could act indirectly by inducing pro-inflammatory responses from the epithelial cells and fibroblasts. Using our current research model, we could simultaneously capture phenotypic changes in multicellular components, suggesting that our 3D respiratory mucosa model is a valuable tool for studying the complicated intercellular communication in the human respiratory mucosa.

Several groups have previously published studies using nasal epithelial cells-on-a-chip. Most of the models recapitulate both the structure and function of the human nose by culturing human nasal epithelial cells [26, 27]. Na et al. generated 3D nasal mucosa-on-a-chip and suggested a possible mechanism for gland-like structural formation in vitro. They found that human microvascular endothelial cells enhance the formation of gland-like structures typically observed in sub-mucosa in vivo. Additionally, Wang et al. developed a human nasal epithelial cells-on-a chip for in vitro testing of formaldehyde toxicity by monitoring cilia beating functions. Even though these previous studies used HNEC, like our model system, their chip design and the number of cell types used were different. They optimized their 3D models based on the purpose of the studies. Our model system was developed to evaluate multicellular responses in the tissue microenvironment using the application of external stimuli to one cell type. We employed three types of cells, including human nasal epithelial cells, human fibroblasts, and human endothelial cells, which were stacked in vertical cell layers to mimic the function and structure of human respiratory mucosa. By exposing the epithelial layer to air pollutants, we triggered cell communication in the chip and analyzed changes in the tissue microenvironment. We believe that our model is useful for understanding multicellular communication in the human respiratory mucosa.

Maintaining tissue integrity is critical in human physiology. Epithelial disruption has been shown to cause various diseases such as allergic rhinitis and Crohn’s disease [9, 10, 12–14]. Enhanced vascular permeability induced by altered integrity of the endothelium is also related to a variety of pathologies [28]. During sepsis, endothelial integrity is disrupted and vascular permeability is increased, which induces an abnormal inflammatory response [29]. As shown in this study, UPM can disrupt the human nasal epithelial integrity by altering the expression of genes related to cell adhesion and inflammation. Previous toxicology studies showed that a nanoparticle is physically able to cut the plasma membrane [30]. Based on previous studies and our results, we expect that UPM can affect human mucosal tissue integrity by both physical and biochemical processes. We also found that UPM induces altered expression of genes that encode for endothelial cell junction proteins. UPM induced pro-inflammatory responses in endothelial cells by upregulating ICAM-1 and disrupting VE-cadherin-mediated junctions. Even though we showed an altered phenotype following exposure to UPM, the detailed underlying molecular mechanism associated with intercellular communication remains to be elucidated.

This in vitro model for human respiratory mucosa is not without its limitations as it lacks other physiological factors, such as immune cells, which are found in the native tissue. Additional tissue-relevant extracellular matrix materials are required for achieving stiffness of the tissue. Therefore, this in vitro organ-on-a-chip and additional tissue-relevant components can be exploited for further development of a physiologically relevant in vitro model of human respiratory mucosa.

## Conclusions

In this study, we demonstrate that the 3D respiratory mucosa model is potentially a valuable tool for the simultaneous evaluation of multicellular responses caused by external stimuli in the human respiratory mucosa. Even though further studies related to the endothelial responses to UPM are required, the results from this study provide significant insight for understanding the effects of UPM on human respiratory mucosa. We also believe that the strategy proposed for evaluating this model will be beneficial for documenting and analyzing multicellular responses to external pathogens in future studies.

## Methods

### Particulate matter preparation

UPM samples (SRM 1648a) were purchased from the National Institute of Standards and Technology in the United States of America. The mean diameter of these particles was 5.85 μm and consisted of 21 metal elements, 4 non-metallic metals, 21 PAHs, and 7 polychlorinated biphenyl congeners. The material was prepared in phosphate-buffered saline (PBS) and sonicated for 20 min directly before experimental use.

### Cell culture preparation and culture of primary human nasal epithelial cells

Human umbilical vein endothelial cells (HUVEC; Lonza Inc., Walkersville, MD, USA), a human lung fibroblast cell line (WI-38; ATCC, Manassas, VA, USA), and normal human lung fibroblasts (NHLF; Lonza Inc.) were cultured in a media recommended by the Lonza Inc. and ATCC. The HUVEC used for the experiments were from passages 6 to 8, NHLF were from passages 5 to 6, and WI-38 cells were from passages 20 to 24.

To harvest the primary human nasal epithelial cells (pHNE), the human inferior turbinate was taken from patients during an augmentation rhinoplasty and treated with dispase for 24 h at 4 °C. Epithelial cells were detached from the turbinate surface by scraping with forceps. The cells were resuspended in DMEM/F12 (Gibco, Dublin, Ireland). This process was repeated twice. The cells were then cultured in airway epithelial cell growth medium (Promocell, Heidelberg, Germany) in 6-well plates. Before obtaining the inferior turbinate, the study protocol was approved by the Institutional Review Board for Human Studies at the Korea University Hospital, College of Medicine, Seoul, Republic of Korea, and all participants provided a written, informed consent (No. 2018AN0061).

### Cell viability assay

Epithelial cells cultured in a 96 well plate and WI-38, HUVECs cultured in 48 well plate were treated with 0, 12.5, 25, 50, 100, 300, and 900 μg/mL of UPM (SRM-1648a, NIST, Gaithersburg, MD, USA). The cells were incubated at 37 °C for 24 or 72 h. After washing them with media containing Cell Counting Kit-8 (CCK-8) (Ez-cytox, Daeillab Service; Seoul, Korea), the cells were incubated for 2.5 h at 37 °C. The CCK-8 utilizes a highly water-soluble tetrazolium salt (WST-8 [2-(2-methoxy-4-nitrophenyl)-3-(4-nitrophenyl)-5-(2, 4-disulfophenyl)-2H-tetrazolium, monosodium salt]). The WST-8 produces formazan dye with absorbance at 450 nm upon reduction in the presence of dehydrogenases in viable cells. Because the absorption of the formazan dye is proportional to the number of living cells, the cell viability was quantified by the absorbance of the formazan dye using a microplate reader (SpectraMax Plus384; Molecular Devices; CA, USA).

### RNA-seq

Cultured epithelial cells were exposed to 100 μg/mL of UPM for 24 hand lysed and purified using QIAzol (Qiagen, Hilden, Germany). To construct complementary DNA (cDNA) libraries with the TruSeq Stranded Total RNA LT Sample Prep Kit (Gold), 1 μg of total RNA was used. The protocol consisted of a polyA-selected RNA extraction, RNA fragmentation, random hexamer-primed reverse transcription, and 100 nt paired-end sequencing using the Illumina HiSeq2500 sequencer (Illumina, San Diego, CA, USA). The raw reads from the sequencer were preprocessed to eliminate low quality reads and adapter sequences before analysis and the reads were aligned to the *Homo sapiens* (hg19) genome assembly using HISAT v2.0.5. Aligned reads were organized to estimate their abundance as FPKM values of genes expressed in each sample using StringTie v1.3.3b. Since the FPKM values have already been standardized for the library size, this value was used for comparative analysis of genes that were differentially expressed between samples.

### Device preparation

The device was designed by modifying the organ-on-a-chip as shown previously [31]. It was fabricated using conventional soft lithography with polydimethylsiloxane (PDMS). The top, middle, and bottom layers of the device were set with PDMS polymer on the master mold and manufactured by Amed, Korea. The diameter of the top and bottom mold was 13 mm and the middle mold diameter was 15.6 mm. The membrane was removed from a 6-well plate with a Transwell polyester (PET) membrane (24 mm diameter, 0.4 μm, Corning Inc. USA). To adhere the membrane to the PDMS layer, bis-amino silane was used to prevent leakage of the cell medium. The removed membrane was first treated with oxygen plasma for 1 min, soaked in 2% bis-amino silane, dissolved in 99% isopropyl alcohol (IPA) at 80 °C for 20 min. It was then immersed in 70% IPA for 30 min, followed by soaking in 70% ethanol for 30 min at room temperature (approximately 25 °C). After the functional group was removed, the PDMS layer was treated with oxygen plasma for 1 min. Finally, the device was assembled as follows: bottom layer, membrane, middle layer, membrane, and upper layer. The device was baked overnight at 70 °C in an incubator. All devices were sterilized using UV light for 24 h.

### Generation of respiratory mucosa-on-a-chip

The PET membranes in the device were coated with fibronectin (20 μg/ml) for 4 h in an incubator at 37 °C. They were washed with PBS and then dried for 24 h in a sterile hood at RT. HUVEC and fibroblasts were seeded at a density of 1X 10^6^ cells /ml in the bottom and middle layers, and the chip was turned over for attachment of cells to the membrane for 4 h at 37 °C. Afterward, the device was turned over, and nasal epithelial cells and fibroblasts were seeded in the top and middle layers, respectively, and incubated at 37 °C. After incubating for 3 days, respiratory mucosa-on-a-chip was ready for the assay.

### Cell tracking and immunofluorescence staining

For tracking the different cell layers in the device, the epithelial cells and HUVEC in each layer were tracked with the CellTracker™ (Thermo Fisher Scientific, Waltham, MA, USA) green, red, and blue dyes. Then the cells that were seeded in each layer were cultured for 2 days before being fixed with 4% paraformaldehyde.

To study changes in the epithelial and endothelial junctions, UPM was exposed to the submerged epithelial cells by loading via a channel for the top layer in the chip. Each cell membrane was stained with ZO-1 (Abcam, Cambridge, MA, USA) and VE-cadherin (Abcam, Cambridge, MA, USA) antibodies using the immunofluorescence staining method. Each cell type that was previously seeded in 24-well cell culture plates was transferred to a glass slide, fixed with freshly 4% paraformaldehyde for 30 min, and permeabilized with 0.5% Triton X-100 at room temperature. Non-specific binding was reduced by blocking the cells with freshly made 1% bovine serum albumin (BSA) in PBS for 1 h at room temperature. Then, the cells were incubated with ZO-1 or VE-cadherin antibody, washed, and incubated with Alexa Fluor 488 conjugated secondary antibody (Abcam, Cambridge, MA, USA). Nuclear staining was performed by DAPI (4′,6-diamidino-2-phenylindole; Sigma, St Louis, MO, USA). ZO-1 and VE-cadherin were visualized using a confocal laser scanning microscope (LSM700; Carl Zeiss; Oberkochen, Germany). Epithelial and endothelial staining was performed using a single chip.

### RT-PCR

Epithelial cells on the chip were treated with 100 μg/mL of UPM (NIST, Gaithersburg, MD, USA). After 24 h, RNA was extracted from the control and UPM-treated cells. To extract the total RNA from the epithelial cells, detached epithelial cells were lysed with QIAzol (Qiagen, Valencia, CA, USA), and the lysates were sequentially treated with chloroform, isopropanol, and ethanol to purify the RNA. cDNA was synthesized from the RNA using a cDNA synthesis master mix (GenDEPOT, TX, USA).

### Real-time PCR

Epithelial cell gene expression was measured by quantitative real-time PCR. The prepared cDNA was amplified and quantified using the SYBR Green master mix (Qiagen, Valencia, CA, USA) with the following primers: GAPDH, forward 5′-GAG TCA ACG GAT TTG GTC GT-3′ and reverse 5′-TTG ATT TTG GAG GGA TCT CG-3′; ZO-1, forward 5′-ACA GTG CCT AAA GCTATT CCT GTG A-3′ and reverse 5′-TCG GGA ATG GCT CCT TGAG-3′; E-cadherin, forward 5′-TGC TCT TGC TGT TTC TTC GG-3′ and reverse 5′-TGC CCC ATT CGT TCA AGT AG-3′; occludin, forward 5′-AGT GTG ATA ATA GTG AGT GCT ATC C-3′ and reverse 5′-TGT CAT ACC TGT CCA TCT TTC TTC-3′; IL-1β, forward 5′-AAA TAC CTG TGG CCT TGG GC-3′ and reverse 5′-TTT GGG ATC TAC ACT CTC CAG CT-3′; IL-6, forward 5′-GTA GCC GCC CCA CAC AGA-3′ and reverse 5′-CAT GTC TCC TTT CTC AGG GCT G-3′; IL-8, forward 5′-ATA AAG ACA TAC TCC AAA CCT TTC CAC-3′ and reverse 5′-AAG CTT TAC AAT AAT TTC TGT GTT GGC-3′; TNF-α, forward 5′-GGA GAA GGG TGA CCG ACT CA-3′ and reverse 5′-CTG CCC AGA CTC GGC AA-3′. Polymerase chain reactions were performed using a real-time thermal cycler system (TP850) (Takara, Shiga, Japan) with 50 cycles of a two-step reaction that consisted of denaturation at 95 °C for 15 s followed by annealing/extension at 60 °C for 45 s. Data were analyzed using the ΔCt method.

### Statistical analysis

Statistical analyses were performed using SPSS for Windows (version 16.0.0; SPSS, Chicago, IL, USA). Data in graphs are expressed as the mean ± SEM of at least three independent determinations. Differences between the control and treated samples were determined using a Student’s *t*-test and the difference between the cytokine-treated groups were calculated using a one-way ANOVA. A *p*-value of < 0.05 was considered significant.

## Data Availability

The datasets supporting the conclusions of this article are included within the article.

## Abbreviations

3D: Three dimensional
AJCs: Apical junctional complexes
COPD: Chronic obstructive pulmonary disease
DEGs: Differentially expressed genes
hNEC: Human nasal epithelial cells
HUVEC: Human umbilical vein endothelial cells
IL-1β: Interleukin-1β
IL-6: Interleukin-6
IL-8: Interleukin-8
IPA: Isopropyl alcohol
NGS: Next-generation sequencing
PAHs: Polycyclic aromatic hydrocarbons
PDMS: Polydimethylsiloxane
PET: Polyester
pHNE: Primary human nasal epithelial cell
PM: Particulate matter
TNF-α: Tumor necrosis factor-α
UPM: Urban particulate matter
VE-cadherin: Vascular endothelial cadherin
ZO-1: Zonula occludens-1
